# Cornual Polyps of the Fallopian Tube Are Associated with Endometriosis and Anovulation

**DOI:** 10.1155/2012/561306

**Published:** 2011-11-29

**Authors:** S. A. AlAsiri, M. Ghahremani, P. F. McComb

**Affiliations:** Division of Reproductive Endocrinology & infertility, Department of Obstetrics and Gynaecology, Faculty of Medicine, BC Women's Hospital and Women's Health Centre, The University of British Columbia, Vancouver, BC, Canada V6H 3N1

## Abstract

*Background*. The relationship between tubal cornual polyps and endometriosis and ovulatory disorders in infertile women is unclear. Our objective was to determine such an association from our database and review the literature. *Methods*. Twenty-two infertile women with tubal cornual polyps were assessed for coexistence of oligoovulation/anovulation and endometriosis with stratification for polyp diameter (large: ≥5 mm diameter, small <5 mm diameter). *Result(s)*. Oligoovulation/anovulation was more prevalent in women with large versus small tubal cornual polyps (*P* = 0.0048). Endometriosis was associated with both large and small polyps. *Conclusion(s)*. This case series confirms the association of tubal cornual polyps with oligoovulation/anovulation and endometriosis in infertile women. This case series is limited by a lack of controls.

## 1. Introduction

**Figure 1:**
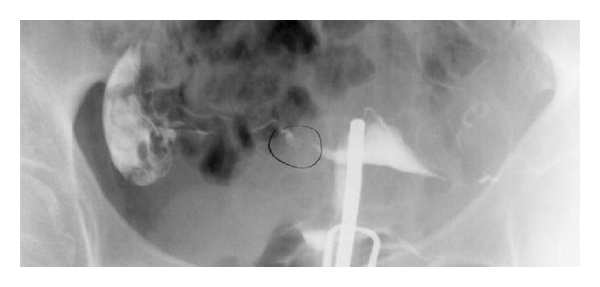
Hysterosalpingography. Circle indicates large right-sided tubal cornual polyp. Note tubal patency.

Tubal polyps, also known as tubocornual or cornual polyps, have been reported sporadically in the gynecologic literature. A common estimate of the prevalence of cornual polyps is 2-3% of the general population who undergo hysterosalpingography (HSG) (Figure 1) [1, 2]. However, the actual reported prevalence varies widely from 1.2% to 33% [3, 4]. Fernstroem and Lagerloef estimated a 10% prevalence of cornual polyps in all women, extrapolated from histological studies of posthysterectomy specimens [3]. It is believed that many polyps are undiagnosed, especially if diminutive in size, due to inaccurate interpretation of HSG imaging and/or an unfamiliarity of the examiner with this condition [2, 3].

**Figure 2:**
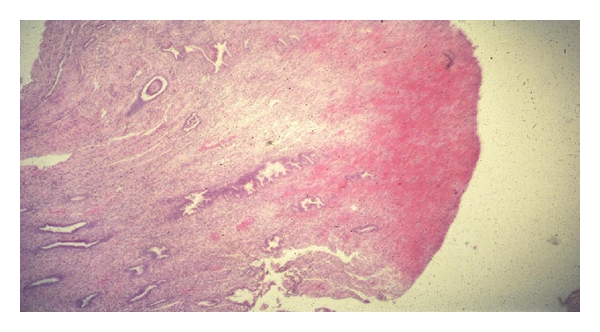
Light microscopy. Hematoxylin and eosin stain of cross-section of a large cornual polyp. Note secretory changes within endometrial glands. Infarction is evident at the right extremity of the polyp. Magnification ×55.

Historically, cornual polyps were first described by Philipp and Huber in 1939 [5]. Cornual polyps are ectopic islands of normal endometrial tissue that arise within the interstitial part of the fallopian tube [6]. They are composed of both endometrial glands and stroma [3, 7, 8] and may manifest secretory change (Figure 2) [3, 5]. However, there is no invasion of the surrounding smooth muscle as occurs in adenomyosis or endometriosis. At HSG, polyps are seen as oval- or round- shaped filling defects, of 3–12 mm in diameter [9]. Conventionally, they are classified as small (<5 mm) and large (≥5 mm) [2]. They usually do not obstruct the fallopian tubes but rather are seen as filling defects within patent tubes at hysterosalpingography [10]. They occur unilaterally or bilaterally [2].

The reported frequency of associated infertility in patients with cornual polyps varies in the literature from 20% to 61.5% [11]. There is inherent bias since women who are infertile are more likely to have an HSG. There have also been sporadic reports of the association of cornual polyps with anovulation and/or endometriosis [1–3, 12]. We therefore wished to determine whether tubal polyps identified on HSG in infertile women in our tubal database are associated with either or both of these conditions. This is especially important given the paucity of data in the literature with respect to these associations and the implications for fertility treatment.

## 2. Materials and Methods

This case series was extracted from the tubal surgery database at the British Columbia Women's Hospital and The University of British Columbia in the period from January 1981 to December 2010. All standardized demographic and clinical data considered to be relevant to tubal infertility are entered prospectively into this ongoing administrative database system (dBase III Ashton-Tate, Culver City, Calif, USA) [13]. Infertility was defined by a period of at least 12 months of unsuccessful attempts at conception. The HSGs were performed predominantly at a single radiology centre and the images reviewed by one of the authors (P.F.McComb). Tubal cornual polyps were diagnosed in infertile women who had undergone an HSG. They were subclassified as large (≥5 mm) and small polyps (<5 mm). All polyps were removed from the cornua by microsurgery at laparotomy. The consecutive pattern of case accrual over time was bimodal. This reflected a belief by one of us (P.F.McComb) that this tubal surgery was of benefit in the early 1980s, and once again in the late 2000s, with an intervening period of equivocation.

 In addition to the HSG, all women underwent a laparoscopy and assessment of ovulatory status. The diagnosis of endometriosis was based upon tissue biopsy or overt visual evidence at the laparoscopy. Anovulation was defined by luteal phase progesterone levels <5 nmol/L and/or by proliferative changes devoid of any secretory activity on endometrial biopsy.

 The database also allowed identification of other potential infertility factors including male factor infertility.

Using the Statistical Package for the Social Sciences for Windows, version 19.0, categorical variables were evaluated by Fisher's exact test. A value of *P* < 0.05 was considered statistically significant.

## 3. Results

Twenty-two women with cornual polyps were referred for infertility evaluation and therapy. Seven women (mean age: 31.7 years; range: 26–40 years) had large cornual polyps (Table 1). Their mean duration of infertility was 46 months (range: 12–84 months). Only one person had neither anovulation nor endometriosis. Four women were anovulatory or oligoovulatory; in each case the cause was polycystic ovarian syndrome. Endometriosis was present in four women. Two women had both anovulation and endometriosis. Five of these women with large polyps also had either intrauterine endometrial polyps and/or prominent endometrial folds.

Fifteen women (mean age: 31 years; range: 25–38 years) had small cornual polyps. The mean duration of infertility was 47 months (range: 15–180 months). None of the 15 women with small tubal cornual polyps had coexisting anovulation; two had coexisting endometriosis. One had stage 1 and the other stage 3 endometriosis according to the revised American Society for Reproductive Medicine (ASRM) [14].

The concurrence of anovulation with large polyps versus small polyps was statistically significant (*P* = 0.004). The association between large polyps versus small polyps and endometriosis approached statistical significance (*P* = 0.053).

## 4. Discussion

This is the first series from a prospective tubal database to study the associations between tubal cornual polyps and anovulation and endometriosis. The limitations of this study are that it was not controlled, blinded, nor randomized. Nevertheless, the strong associations of these conditions with large cornual polyps are clinically meaningful.

Tubal cornual polyps may cause infertility in a variety of ways.

The pseudostratified endosalpinx of the intramural tube is characterized by a relative abundance of secretory cells [15]. This segment has three muscle layers: an outer spiral-longitudinal layer, which blends with the myometrium, and circular and inner longitudinal layers which penetrate as a distinct core deep into the myometrium [16]. The luminal diameter is 0.5 mm or less. The inherent intramural myosalpingeal contractility is similar to that of the neighboring isthmus, but acts upon a narrower and more deviating lumen than that of the isthmus [16]. By modulation of this contractility, the intramural portion of the fallopian tube initially retains the embryo within the isthmus of the fallopian tube for up to 3 days after ovulation and then releases the embryo into the endometrial cavity.

A large cornual polyp can perturb the structure and physiology of the intramural oviduct by dilatation of the lumen severalfold, alteration of muscular contractility, and/or the presence of the ectopic endometrium within the tube (with mucosa and glandular secretion that differs from that of the pseudostratified endosalpinx). We have also observed infarction of the distal extremity of these polyps at surgery (Figure 2); this may also interfere with embryo nurture and transport. Infarction of large cornual polyps has also been reported by Bret and Grépinet [11]. It should be noted that cornual polyps do not appear to prevent the passage of contrast medium through the fallopian tube [10]. 

 The reported frequency of an association of tubal polyps with infertility varies from 27% to 62% [2]. However, it remains unclear as to whether there is a direct causal relationship. This becomes especially tenuous when endometriosis and anovulation are potentially at play.

The association between tubal cornual polyps and anovulation and endometriosis has previously been reported in the literature [11] (Table 2). The polyp dimension is not always stated so that it is difficult to arrive at any consensus. In our series it is the large (bilateral) polyps that associate strongly with anovulation and endometriosis.

Zenisek [12] concluded that tubal polyps were caused by endometrial hyperplasia and found hyperplastic eutopic endometrium in all 10 of his tubal polyp cases. Five of the 17 patients with polyps reported by Fernstroem and Lagerloef [3] had glandular cystic hyperplasia (2 women) or polypoid hyperplasia (3 women) on endometrial biopsy. David et al. [2] found that 39% of women who had tubal polyps were anovulatory. Anovulation was the most common attributable cause of infertility in their group. A higher prevalence of anovulation in conjunction with cornual polyps has also been reported in two French studies [11, 17]. Furthermore, McLaughlin et al. [18] and Stangel et al. [1] each reported a woman with cornual polyps who was anovulatory. Logically, some have proposed that treatment of the coexisting anovulation would significantly increase the prospects for conception [1].

An association between cornual polyps and endometriosis has also been previously suggested. A summary of reported cases is shown in Table 2. Historically, in 1939, Philipp and Huber postulated that cornual polyps may cause endometriosis by discharging and spreading endometrial tissue via the fallopian tubes [5]. Fernstroem and Lagerloef reported four cases of ovarian and peritoneal endometriosis observed at laparotomy of their 26 cases of tubal polyps [3]. They concluded that there is presumptive evidence that women with tubal polyps have an increased tendency to develop ovarian or peritoneal endometriosis. Lisa and coworkers studied the intramural portions of the fallopian tubes in 300 posthysterectomy uteri [4]. They were the first to show that cornual polyps are composed of endometrial tissue. Furthermore, they documented associated endometriosis at other pelvic sites. These authors concluded that the presence of endometrial tissue within the tubes appeared to be a normal developmental phenomenon. All 5 patients with tubal polyps reported by Gillett [8] had pelvic endometriosis. In the series reported by Gordts et al. [7], 41% of the 44 patients with cornual polyps who underwent laparoscopy had endometriosis. The anovulatory infertile woman with bilateral cornual polyps that McLaughlin [18] reported also had stage 2 endometriosis. Glazener et al. found no association with either endometriosis or anovulation, but it is unclear as to whether all their women with cornual polyps underwent laparoscopy to diagnose endometriosis and/or had assessment of ovulation [19].

 We hypothesize that the coexistence of anovulation and endometriosis may lead to cornual polyp formation because both conditions are associated with proliferation of endometrium and/or formation of endometrial polyps. In our series, 5 of the 7 women with large cornual polyps had either endometrial polyps and/or prominent endometrial folds.

Some anovulatory syndromes, such as polycystic ovarian syndrome, predispose to estrogen-stimulated endometrial proliferation and hyperplasia [20, 21].

Women with endometriosis often form endometrial polyps [22]. Within the eutopic endometrium of women with endometriosis, there are multiple biochemical derangements. These include an increase in cycloxygenase-2 (COX-2) activity and aromatase activity, with overproduction of estrogen, prostaglandins, and cytokines [23]. Cellular proliferation is increased, and levels of apoptosis-related proteins are decreased [24]. Recently, it has been shown that endometriosis can be reliably diagnosed by the detection of increased nerve fiber density by immunohistochemistry in eutopic endometrium throughout the menstrual cycle [25, 26]. Such functional alterations, or abnormalities, in eutopic endometrium may predispose to endometrial polyp formation, and to cornual polyp formation.

In conclusion, the results of our case series confirm previous reports that infertile women with cornual polyps of the fallopian tube are more likely to have associated endometriosis and/or anovulation. It is possible that proliferative endometrial development and formation of endometrial polyps that may attend anovulation and endometriosis also predispose to the formation of cornual polyps. However, future studies with larger sample sizes are needed to better delineate this association.

Our findings of multiple infertility factors associated with tubal cornual polyps suggest two alternative therapies. Either IVF therapy and/or microsurgery to remove both the cornual polyps and ablate endometriosis, followed by induction of ovulation in anovulatory women.

## Figures and Tables

**Table 1:** Clinical characteristics of infertile women with large cornual polyps.

Case	Age in years	Duration of infertility (months)	Polyp(s) size (mm)	Uterine cavity (HSG)	Anovulation	Endometriosis	ASRM stage endometriosis
1	34	59	Bilateral 3 × 8	Normal	Yes	No	N/A^a^
2	27	12	Bilateral 8 × 4	Normal	No	Yes	1
3	37	60	Right: 4 × 3 Left: 5 × 2	Polyp	No	Yes	1
4	40	84	Right: 4 × 2 Left: 6 × 4	Endometrial polyp	Yes	Yes	1
5	26	20	Right: 9 × 2 Left 12 × 3	Prominent endometrial folds	Yes	Yes	1
6	28	59	Bilateral 13 × 4	Endometrial polyps, endometrial fold	No	No	N/A^a^
7	30	28	Right: 6 × 2 Left: 5 × 2	Prominent endometrial fold	Yes	No	N/A^a^

^a^ N/A: not applicable.

**Table 2:** Summary of published reports of the association of cornual polyps with anovulation (ovulatory dysfunction) and endometriosis.

Study	Year	Number of cases reported	Number of cases with anovulation (ovulatory dysfunction) and/or endometrial hyperplasia	Number of cases with endometriosis
Zenisek [12]	1959	10	10	0
Fernstroem and Lagerloef [3]	1964	17	5	4
David et al. [2]	1981	54	3	0
Gaudefroy et al. [17]	1970	47	10	0
McLaughlin [18]	1984	1	1	1
Glazener et al. [19]	1987	31	0	0
Stangel et al. [1]	1981	1	1	0
Philipp and Huber [5]	1939	4	0	4
Gordts et al. [7]	1983	52	Unstated	18
Gillett [8]	1989	5	0	5
